# Prevention of substance abuse in youth: How social norms approach can help

**DOI:** 10.1192/j.eurpsy.2021.178

**Published:** 2021-08-13

**Authors:** E. Sönmez

**Affiliations:** Department Of Psychiatry, Zonguldak Caycuma State Hospital, Istanbul, Turkey

**Keywords:** social norms, youth, alcohol use, prevention

## Abstract

Drug and alcohol use in adolescence is a major global public health concern. Adolescence is the highest risk period for the initiation of drinking and substance use. Since the 90s, a growing body of evidence has indicated the influence of peers’ behaviours and attitudes in the development of youth tobacco, alcohol and drug use. Based on these studies, the social norms approach has been developed, mainly applying to the Western countries. The approach argues that how a student perceives his/her peers’ health-related behaviours and attitudes (such as substance/alcohol use) does have an influence on his/her own behaviour, and negative behaviours are generally overestimated among peers. Correcting these misperceptions may contribute to the prevention of youth substance/alcohol use. The applicability and discussions on the social norms approach will be presented in this talk, with two example studies. Both studies are conducted in Turkey, which is ranked lowest in terms of alcohol use and related problems among World Health Organization Europe zone countries, despite and increasing trend in use over the past decades. In both university and high-school samples, we found that students’ misperceptions about higher peer tobacco and alcohol use facilitated their own alcohol use. We conclude that targeting social norms may be part of a generalized preventive approach with regards to drug use and is of universal value. References: 1. SÖNMEZ, E. & AKVARDAR, Y. 2015. A Social Norms Approach to Substance Abuse Prevention in Youth “The more I think you drink, the more I drink”. Bağımlılık Dergisi-Journal of Dependence, 16, 86-94 (Turkish) 2. GÜNDÜZ, A., SAKARYA, S., SÖNMEZ, E., ÇELEBI, C., YÜCE, H. & AKVARDAR, Y. 2019. Social norms regarding alcohol use and associated factors among university students in Turkey. Archives of Clinical Psychiatry (São Paulo), 46, 44-49.

**Disclosure:**

No significant relationships.

